# Mr. Bartlett, on Opium in Intermittent Fever

**Published:** 1804-07-01

**Authors:** Michael Bartlett

**Affiliations:** Finsbury Dispensary, St. John's Square


### *To the Editors of the Medical and Physical Journal.*

GENTLEMEN,

CASES of intermittent fever, from their being of rare occurrence in the practice of this Metropolis, seem not to have excited sufficient attention to their mode of treatment. When they do occur, they are imported from marshy countries, where the disease is endemic; and bark, in one form or other, is considered the specific remedy.

Notwithstanding that this disease is produced by marsh miasma, there are many other causes which may *predispose* the body to be acted upon by this unwholesome effluvia, such as cold and moist atmosphere, feeding upon acid fruits, crude and watery vegetables, low living in general; and, in short, every thing that tends to impoverish the fluids and impair the tone of the solids, favour very much the production of this complaint; a sufficient reason why they are found to be more frequent in the former classes of society than among the rich and affluent.

Agues are much more violent in their paroxysms in the country than they are in town, our noxious smokey atmosphere being, as it were, an antidote to this disorder. It is with a view to draw the attention of medical men more particularly to the use of opium in this complaint, that I am induced to publish the two following cases, where it will be seen to have produced the most striking and beneficial effect.

John Gash, a young man of about twenty years of age, of a pallid countenance, and weak lax fibre, became a patient of the Dispensary the seventeenth day of April, for the cure of an intermittent of the quotidian type, which he had contracted during a journey into Essex, where he had not remained eight and forty hours before he was siezed with this complaint; the bark was prescribed for him in doses of ʒj. omni 2d. hora sumd. which he continued taking until the 23d of April following, without any material alteration or benefit in his complaint; when I had occasion to see him in the absence of the attending physician, and ordered him to take the following draught an hour antecedent to the recurrence of the cold fit.

℞. Tinct. Opii. gt. xxxv. Zinzib. ʒj. Aq. menth. pip. ℥jß. M. ft. haust.

This produced a very favourable effect, for the fit was not nearly so violent nor long in its duration as it had been before. On the following day the draught was repeated with the addition of five drops of tinct. opii; the fit was scarcely perceptible. On the third day the draught was again repeated, when the fit did not return, nor has he felt any thing of it since.

Thomas Bentley, ætat. 23, of a habit of body similar to that of the former patient, applied to the Dispensary for the cure of a tertian ague. He had been taking the bark for nearly a fortnight (when I saw him) without any material benefit. I gave him the same draught which had produced so good an effect in the before mentioned case, and the effect here was equally striking and beneficial, the second dose having removed his complaint altogether.

Bark, therefore, although it, with an almost infallible certainty, cures the disease, ought not strictly to be regarded as a *specific* in this complaint, as a specific, properly so called, not merely cures, but is the only thing which *can* cure the malady to which it is applied.

The practice of giving opium in intermittent fever is neither singular nor novel, it having been adopted by medical practitioners at least half a century ago; in some old authors which I have read, this practice is mentioned, particularly by Dr. Berryatt, a French physician, who avers that he has found Dr. Sydenham's liquid laudanum more infallible in the cure of intermittent fever than even the bark itself, a single dose for the most part sufficing; he gave it to the patient in bed, an hour before the cold fit was expected, in doses of twenty-five to thirty drops.

Dr. Lind, an intelligent physician, who appears to have paid a great deal of attention to this disease, speaks very highly as to the effect of this medicine, when given during the hot fit. But, surely, it appears more consistent with the theory of the cold fit depending upon spasm, that to apply the remedy just before its attack, would be more likely to alleviate its violence and shorten its duration, and thus mitigate the violence, and abridge the continuance of the succeeding reaction. I am, &c.

MICHAEL BARTLETT.

*Finsbury Dispensary, St. John's Square, May 29,* 1804.

